# Highly Stretchable Shape Memory Self-Soldering Conductive Tape with Reversible Adhesion Switched by Temperature

**DOI:** 10.1007/s40820-021-00652-0

**Published:** 2021-05-11

**Authors:** Mengyan Wang, Quan Zhang, Yiwen Bo, Chunyang Zhang, Yiwen Lv, Xiang Fu, Wen He, Xiangqian Fan, Jiajie Liang, Yi Huang, Rujun Ma, Yongsheng Chen

**Affiliations:** 1 School of Materials Science and Engineering, National Institute for Advanced Materials, Tianjin Key Lab for Rare Earth Materials and Applications, Nankai University, Tongyan Road 38, Tianjin, 300350 People’s Republic of China; 2 State Key Laboratory and Institute of Elemento-Organic Chemistry, Centre of Nanoscale Science and Technology and Key Laboratory of Functional Polymer Materials, College of Chemistry, Nankai University, Weijin Road 94, Tianjin, 300071 People’s Republic of China

**Keywords:** Shape memory performance, Self-soldering conductive tape, Reversible adhesion, Stretchable electronics

## Abstract

**Highlights:**

- Shape memory self-soldering tape used as conductive interconnecting material.
- Perfect shape and conductivity memory performance and anti-fatigue performance.
- Reversible strong-to-weak adhesion switched by temperature.

**Abstract:**

With practical interest in the future applications of next-generation electronic devices, it is imperative to develop new conductive interconnecting materials appropriate for modern electronic devices to replace traditional rigid solder tin and silver paste of high melting temperature or corrosive solvent requirements. Herein, we design highly stretchable shape memory self-soldering conductive (SMSC) tape with reversible adhesion switched by temperature, which is composed of silver particles encapsulated by shape memory polymer. SMSC tape has perfect shape and conductivity memory property and anti-fatigue ability even under the strain of 90%. It also exhibits an initial conductivity of 2772 S cm^−1^ and a maximum tensile strain of ~ 100%. The maximum conductivity could be increased to 5446 S cm^−1^ by decreasing the strain to 17%. Meanwhile, SMSC tape can easily realize a heating induced reversible strong-to-weak adhesion transition for self-soldering circuit. The combination of stable conductivity, excellent shape memory performance, and temperature-switching reversible adhesion enables SMSC tape to serve two functions of electrode and solder simultaneously. This provides a new way for conductive interconnecting materials to meet requirements of modern electronic devices in the future.
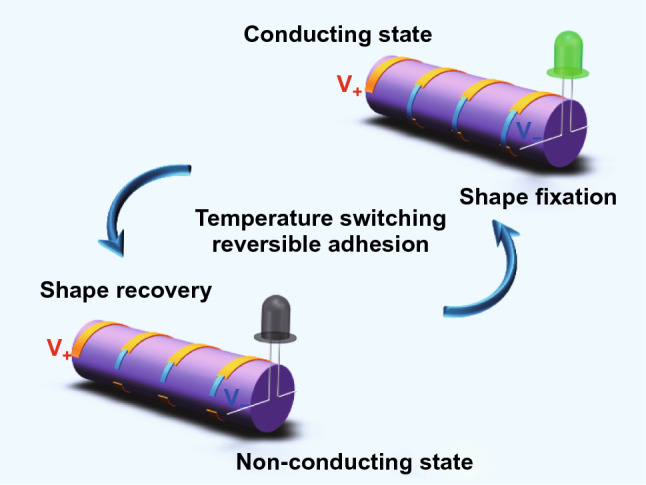

**Supplementary Information:**

The online version contains supplementary material available at 10.1007/s40820-021-00652-0.

## Introduction

In recent decades, modern electronic devices are developing toward integration, miniaturization, flexibility, scalability and biocompatibility [1–4]. Extensive studies on modern electronic devices focus on the functional elements, such as transistors [5, 6], sensors [7–11], electrodes [12–14], capacitors and batteries [15, 16], however, less attention has been paid to the related electronic interconnecting materials [17]. Among conventional electronic interconnecting materials, tin-based alloy solder and conductive adhesive are commonly used in modern electronic devices. In order to form a permanent conductive bond between the two elements, tin-based solder is first melted at a temperature higher than its melting temperature and then re-solidified at the connecting interface after cooling. High processing temperature may damage the substrate and temperature-sensitive elements. In contrast, epoxy-based commercial silver paste can form a good conductive way between various electronic elements after solvent volatilization, where it is not necessary for high temperature treatment [18]. Even so, the solvent in the paste may corrode the substrate and electronic elements. Meanwhile, the packaging structure with reduced volume for microelectronics has to face the reliability issue from the microscopic damage accumulation [19, 20]. Moreover, conventional electronic interconnecting materials are generally un-stretchable and may either crack at a strain as low as 1% or deform plastically at a strain greater than a few percent. These rigid and un-stretchable electronic interconnecting materials cannot meet the flexible requirements of wearable electronics. Therefore, it is imperative to develop new conductive interconnecting materials which possess stable conductivity, high stretchability, lower using condition and strong adhesion appropriate for modern electronic devices [18].

The most direct method to develop conductive interconnecting materials is to improve the existing interconnecting materials according to the demands of modern electronic devices. For example, tin-bismuth alloy solder with lower melting temperature can be used at processing temperature below 200 °C [21–23]. However, the defects such as segregation behavior and low adhesive strength hinder the application of tin-bismuth alloy solder [24, 25]. In order to prevent the matrix and electronic components from being corroded by solvents, adhesives with different solvents were fabricated toward the specific needs of conductive materials [26–28]. Among them, the newly developed waterborne adhesive has a wider range of applications, while the oxidation and short circuit problems caused by water vapor need more attention [29–31]. In addition to the above two types of conductive interconnecting materials, researchers have also tried to develop various dry adhesives [32, 33]. Carbon nanotubes (CNTs) with hierarchical structures can provide an outstanding and stable adhesion for thermal and/or electric management [34, 35]. However, the relative research still stays in the laboratory due to the difficulties in the development of CNTs with ideal hierarchical structures. Another kind of dry adhesive realizes reversible adhesion based on shape memory effect [36–38]. The poor conductivity of shape memory polymer (SMP) is the biggest challenge to apply this dry adhesive for circuit repair. However, the characteristics of existing conductive interconnecting materials, such as high processing temperature, rigidness and corrosivity, are not compatible with the development of miniaturization, integration and flexibility of modern electronic devices. This mismatch limits the development of modern electronic devices. In order to fully play the role of modern electronic devices, it is particularly important to develop new conductive interconnecting materials.

In this work, we reported highly stretchable shape memory self-soldering conductive tape (SMSC tape) with reversible adhesion switched by temperature. The SMSC tape exhibited an initial conductivity of 2772 S cm^−1^ and a maximum tensile strain of ~ 100%. The maximum conductivity could be increased to 5446 S cm^−1^ by decreasing the strain to 17%. It also had a perfect shape memory performance with strain fixation ratio and recovery ratio of 100%. Meanwhile, there was no obvious degradation of shape memory performance after repeated stretching-recovery cycles. Thanks to the temperature induced shape recovery property, SMSC tape easily enabled reversible strong to weak adhesion transition. The combination of stable conductivity, excellent shape memory performance and reversible adhesion gives SMSC tape versatility to independently finish the collaborative work of multiple materials or cannot be completed by existing interconnecting materials.

## Experimental

### Materials

Silver acetate (99%) was obtained from Adamas-beta, saturated ammonia solution (AR 25–28%), formic acid aqueous solution (AR 88%) and stearyl acrylate (SA) (> 96.0% GC) were purchased from Aladdin. Urethane diacrylate (UDA) (CN9021 NS) was acquired from Sartomer. Benzoyl peroxide (BPO) (98%) was purchased from MERYER. They were not purified before use.

### Synthesis of Silver Nanoparticles (Ag NPs)

Ag NPs were prepared by thermal reduced method following a previous report [39]. Silver acetate (1 g) was dissolved in saturated ammonia solution (10 mL) by stirring at room temperature. After that, formic acid aqueous solution (1 mL) was added dropwise with continuous stirring. During this period, the solution turned muddy and then transparent. The transparent solution stood 12 h to settle down large Ag NPs. Then, the supernatant was transferred to a dry culture dish and heated at 70 °C for 1 h. Further, the product was fully dried at 50 °C in the air. The dried product was washed by centrifugation using deionized water to remove impurities. Finally, the precipitation was fully dried at 60 °C in the air.

### Preparation of SMSC Tape

SMSC tape was prepared by in situ formation of SA-UDA copolymer in blended composite under the condition of anaerobic heating. SA, UDA, BPO, and thermal reduced Ag NPs were proportionally mixed by vortex mixing using as the precursor of SMSC tape. After that, the precursor was degassed in a vacuum oven at 50 °C. Then, the precursor was injected between a pair of warm glass sheets spaced with Kapton tape of 280 μm. The precursor was subsequently cured at 80 °C for 80 min. Finally, the film was cooled to room temperature and gently peeled off from the glass sheets. The film can be further cut into required shape.

### Characterization and Evaluation

The morphology of Ag NPs and SMSC tape was observed by SEM (JSM-7800F, JEOL). The structural information of Ag NPs and SMSC tape was characterized by XRD (Rigaku Smart Lab SE). DSC analysis was performed by the thermogravimetric analyzer (Netzsch STA 449F5) with a heating rate of 0.5 °C min^−1^. The tensile strain of SMSC tape was achieved by a home-made stress–strain test system, which was connected with a Keithley 2182A/6221 system to record all the electric signals in real-time. Two quantities, namely strain fixation ratio (*R*_*f*_) and strain recovery ratio (*R*_*r*_), were used to evaluated the shape memory performance of SMSC tape. They were, respectively, calculated as following:

1 $$R_{f} = \frac{{L_{f} - L_{0} }}{{L_{s} - L_{0} }} \times 100\%$$

2 $$R_{r} = \frac{{L_{s} - L_{r} }}{{L_{s} - L_{0} }} \times 100\%$$

where *L*_0_, *L*_*s*_, *L*_*f*_, and *L*_*r*_ are original length, tensile length, temporarily fixing length and final recovery length of SMSC tape, respectively. The shear adhesive strength of SMSC tape was also measured using home-made stress–strain measuring system. SMSC tape (5 × 5 mm^2^) is sandwiched between two copper (Cu) plates by pressing (preloading force of ~ 4 kPa) at the heating state. Two Cu plates are fixed by the clamps in the measuring system, where the adhesive interface is parallel to the tensile direction. The load is recorded in real-time with the increase in distance between two clamps. The recorded maximum load before separation of two Cu plates is the corresponding shear strength. The output voltage and current of TENG made of SMSC tape were measured with a digital oscilloscope (DS1104, RIGOL) and a low noise electrometer (6514 system electrometer, Keithley).

## Results and Discussion

### Tape Preparation and Characterization

**Fig. 1:**
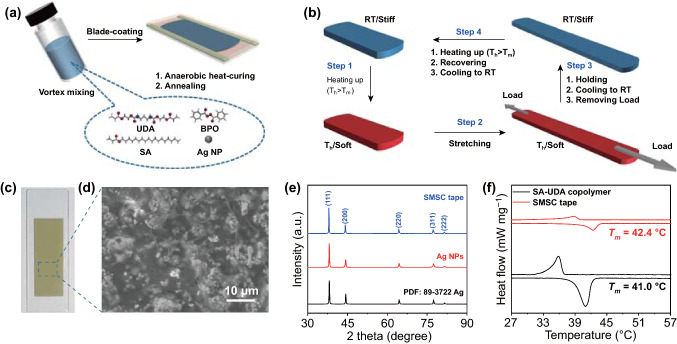
Preparation and Characterization of SMSC tape. **a** Schematic illustration of the preparation process. **b** The shape memory effect of SMSC tape. **c** Optical image of as-prepared SMSC tape. **d** Cross-sectional morphology of SMSC tape. The scale bar is 10 μm. **e** XRD patterns of Ag NPs, SMSC tape and the powder diffraction file (PDF) of 89–3722 Ag.** f** DSC diagrams of SMSC tape (red line) and pure SA-UDA copolymer (black line)

SMSC tape is prepared by anaerobic curing of well blended precursors, including SA, UDA, BPO, and Ag NPs (Figs. S1–S3). Under the condition of anaerobic heating, BPO induces radical reaction between SA and UDA to form the SMP in situ in composite. Meanwhile, the uniformly distributed Ag NPs construct a conductive network in the polymer. Annealing treatment further improves the electric conductivity of SMSC tape (Fig. 1a). At room temperature, SMSC tape can be cut into the required size. When it is heated above the melting temperature (*T*_*m*_), it is easily deformed to the desired shape. The temporary shape is maintained at room temperature until it is reheated to the melting temperature (Fig. 1b). Figure 1c is a digital optical image of an as-prepared SMSC tape on the glass substrate. The cross-sectional scanning electron microscope (SEM) image shows that Ag NPs are uniformly dispersed in the cross-linked SA-UDA SMP (Fig. 1d). The diameter center of the synthesized Ag NPs is ~ 300 nm. The X-ray diffraction (XRD) pattern is almost the same as the standard powder diffraction file (PDF) of 89–3722 Ag, which indicates that the synthesized Ag NPs have high crystalline. After annealing treatment, the crystallinity of Ag NPs do not change significantly (Fig. 1e). Compared with pure SA-UDA SMP, the melting temperature of SMSC tape is slightly higher and the melting enthalpy (Δ*H*_*m*_) is greatly reduced. The former shows that Ag NPs do not decrease the crystallinity of SA-UDA copolymer, while the latter is due to the low specific heat of Ag NPs (Fig. 1f and Table S1).

### Electrical and Mechanical Properties

**Fig. 2:**
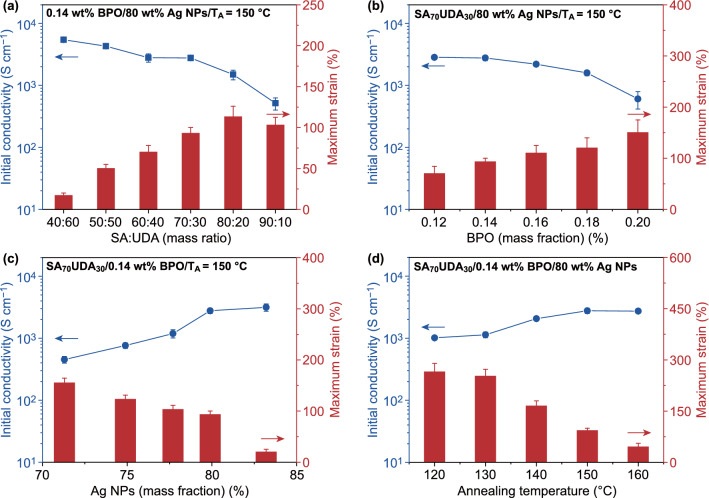
Effects of four parameters on initial conductivity and maximum strain of SMSC tape at 60 ℃. **a** The mass ratio between SA and UDA. **b** The content of BPO. **c** The content of Ag NPs. **d** Annealing temperature

Four comparative experiments in Fig. 2 analyzed the initial conductivity and maximum strain at 60 °C of SMSC tape synthesized by different formulations in detail. The weight ratio between SA and UDA is a key parameter that affects the conductivity and maximum strain of SMSC tape. As shown in Fig. 2a, the initial conductivity decreases with the mass ratio between SA and UDA from 40:60 (SA40UDA60) to 90:10 (SA90UDA10). Meanwhile, the maximum strain increases first and then decreases, where the inflection point is 80:20 (SA80UDA20). SA is less dense than UDA. This means that the same weight of SA is added into the SMSC tape instead of UDA to keep the total mass fraction of polymer constant in value, but the actual volume fraction of the polymer gradually increases as the SA content increases. The increase in polymer volume, namely the decrease of Ag NPs content, results in the decrease of SMSC tape conductivity. In addition, the maximum tensile strain of pure SMP made of SA and UDA decreases with the increase in SA content [40]. For the SMSC tape composed of polymer with 90:10 mass ratio of SA and UDA, the maximum strain decreases due to the decrease in intrinsic maximum strain of pure SMP, although the actual volume fraction of polymer (90:10 mass ratio) in SMSC tape is bigger than that with 80:20 mass ratio. To meet the requirements of conductivity and stretchability simultaneously, the mass ratio between SA and UDA is kept at a fixed value of 70:30 (SA70UDA30) to optimize other components and treatment conditions in SMSC tape.

Another important factor to consider is the content of BPO. Here, BPO acts as radical initiator to promote radical reaction of SA and UDA under the condition of anaerobic heating. Thus, more BPO can induce more dramatic polymerization between SA and UDA in the same curing period of time. In other words, more polymer chains with high molecular weight are formed in the SMSC tape. The existence of these long-chain molecules is conducive to the ductility of SMSC tape. On the other hand, they hinder the formation of conductive paths in SMSC tape. As shown in Fig. 2b, the initial conductivity of the SMSC tape decreases with the increase of BPO content, but the maximum strain gradually increases.

The content of Ag NPs directly determines the conductivity of the SMSC tape. The more Ag NPs are incorporated into the composite, the higher conductivity is reached. Figure 2c shows that the initial conductivity significantly increases with the increase in Ag NPs content, while the maximum strain decreases. Under the premise of sufficient Ag NPs content, the annealing treatment can improve the electric contact between adjacent Ag NPs. As the annealing temperature (*T*_*A*_) rises, the initial conductivity increases, but the maximum strain decreases (Fig. 2d). In general, there is a trade-off between the initial conductivity and the maximum tensile strain of the SMSC tape. In essence, it is a balance between conductive network that improves conductivity and interaction of molecule chain for increasing stretchability.

### Shape-Memory Performance

**Fig. 3:**
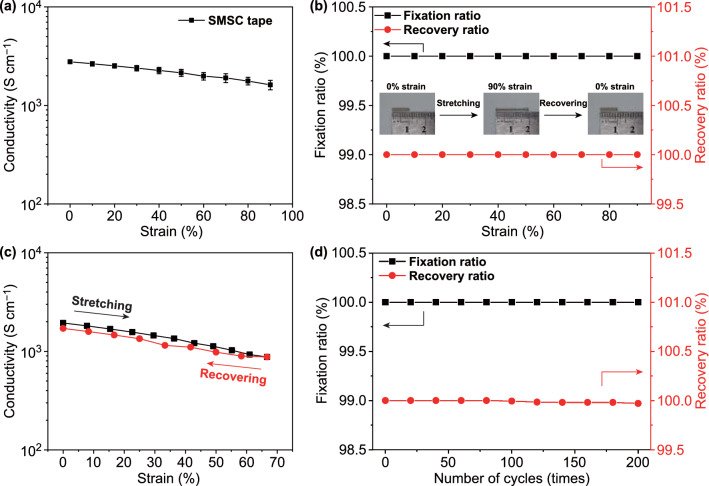
Shape-memory performance of SMSC tape. **a** Conductivity of SMSC tape at room temperature as a function of tensile strain. **b** *R*_*f*_ and *R*_*r*_ of shape fixing and recovery of SMSC tape at different tensile strains. **c** A cyclic conductivity-strain test is carried out using SMSC tape at maximum strain of 66.7%. **d** *R*_*f*_ and *R*_*r*_ of cyclic shape fixing and recovery of SMSC tape at tensile strain of 50%. The mass ratio between SA and UDA is 70:30. The mass fraction of Ag NPs and BPO is 80% and 0.14%, respectively. The annealing temperature is 150 ℃

SMSC tape containing SA-UDA copolymer encapsulated Ag NPs exhibits almost perfect shape memory performance. Here, 150 °C annealed SMSC tape containing 19.86 wt% SA_70_UDA_30_ copolymer, 80 wt% Ag NPs and 0.14 wt% BPO is used to demonstrate the recovery capacity of conductivity and shape memory effect. It has an initial conductivity of 2772 S cm^−1^ and a maximum strain of ~ 100%. The conductivity gradually decreases with the tensile strain and it is still 1618 S cm^−1^ at 90% (Fig. 3a). At 60 °C, the elastic modulus of SMSC tape is 0.36 MPa and the maximum tensile stress before fracture is only 0.20 MPa, both of which are much lower than those of shape memory alloy. In contrast, the elastic modulus and the maximum tensile stress of SMSC tape at room temperature are 52.2 and 3.7 MPa, respectively (Fig. S4 and Table S1) [41]. In the heated state, such a low elastic modulus and maximum tensile stress make the shape of the SMSC tape easy to change without a large external load. The strong mechanical strength at room temperature enables SMSC tape to resist the external impact. Strain fixation ratio (*R*_*f*_) and strain recovery ratio (*R*_*r*_) are two important quantities for evaluating shape-memory performance. They are calculated using equations described in experimental methods. The former has the ability of switching segments (SA) to fix the temporary shape, while the latter is used to describe the ability of the shape-memory segment (UDA) to memorize the permanent shape. Figure 3b shows *R*_*f*_ and *R*_*r*_ of SMSC tape as a function of tensile strain. Under different strains from 10 to 90%, the SMSC tape shows perfect ability of shape-fixation and shape-recovery, namely almost 100% *R*_*f*_ and 100% *R*_*r*_ (Video S1). The insets in Fig. 3b give a visual representation of the perfect shape-fixation at 90% strain and shape-recovery.

SMSC tape shows good recovery capacity of conductivity thanks to its excellent shape memory performance. As shown in Fig. 3c, there is no obvious hysteresis in conductivity during a stretching and releasing process. With the repeating stretching-releasing cycles, the conductivity of SMSC tape decreases and gradually becomes a stable value, when maximum strain is 20% (Fig. S5). Furthermore, we evaluated the fatigue resistance capability in shape memory performance of SMSC tape (Fig. 3d). When the maximum strain is 50%, *R*_*f*_ has always maintained 100% after 200 cycles, which indicates that the SMSC tape has near perfect anti-fatigue performance in shape-fixation ability. Meanwhile, when the number of cycles is less than 100 times, *R*_*r*_ is also 100%. And from 100 to 200th cycles, the degradation of *R*_*r*_ decreases from 100 to 99.93%. However, due to the recoverable conductivity and near perfect shape memory performance, SMSC tape can be easily reused many times in practice.

### Temperature-Dependent Reversible Adhesion

**Fig. 4:**
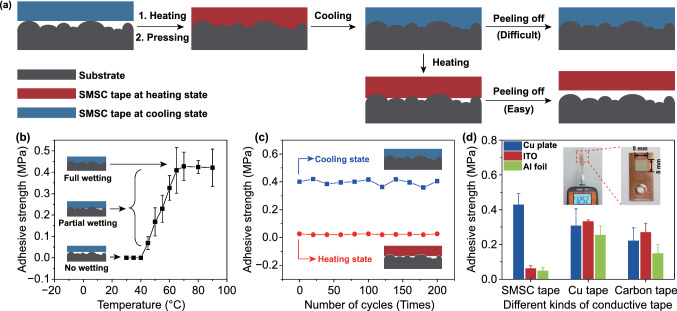
Reversible adhesion of SMSC tape switched by temperature. **a** Schematic illustration of adhesion reversible process of SMSC tape switched by temperature. **b** Shear adhesive strength between SMSC tape and Cu plate at room temperature switched by different heating temperature. Inset is the wetting state of SMSC tape at cooling state on rough substrate with the different heating temperature. **c** Reversible shear adhesive strength of SMSC tape between heating state and cooling state as a function of the number of cycles. The switching temperature is 70 ℃. **d** Shear adhesive strength of SMSC tape and two kinds of commercial conductive tape on various conductive surfaces. The inset is a digital image illustrating the adhesion force of SMSC tape on the Cu plate

Besides good conductivity and perfect shape memory performance, SMSC tape also shows temperature-dependent reversible adhesion. SMSC tape is rigid at room temperature, which is unable to obviously wet the surface. Thus, it has no adhesion ability. When the SMSC tape is heated above the melting temperature, the SMSC tape softens and gradually wets the surface under a moderate pressure, resulting in sharp increase in adhesion upon cooling (Fig. 4a). Figure 4b shows the shear adhesive strength of SMSC tape in the cooling state as a function of heating temperature. When the temperature is equal to or lower than 40 °C, the shear adhesive strength is almost zero. Once the heating temperature is higher than 45 °C, which is larger than the melting temperature, the shear adhesive strength of SMSC tape increases significantly and reaches to the highest value (~ 0.43 MPa) after heating at 70 °C. As the heating temperature increases further, shear adhesive strength gradually decreases due to the excessive softening of SMSC tape (Fig. S6). We also measured the shear adhesive strength of SMSC tape (only 0.02 MPa) at heating state (70 °C), which was significantly lower than that in cooling state. The shear adhesive strength evolution of SMSC tape during 200 cycles of repeated attaching-peeling with 70 °C heating temperature is shown in Fig. 4c. There is no obvious increase or decrease in shear adhesive strength under heating and cooling state. Strong shear adhesive strength in the cooling state can meet the requirement of connection between two terminals, while the weak shear adhesive strength in the heating state make it easy to peel off the SMSC tape. Meanwhile, due to reversible and stable adhesion, SMSC tape is not disposable but recyclable. SMSC tape can also provide extraordinary adhesion on various substrates, especially 0.42 MPa on Cu plate, which is even stronger than that of commercial conductive tape (Fig. 4d). Different from commercial adhesives, the stickiness of SMSC tape is the embedded structure between SMSC tape and the substrate in essence, rather than the molecular interaction between the macromolecules in the adhesive and the target materials [37]. Thus, the surface roughness of the object is an important factor influencing the adhesive strength of SMSC tape on the object. Compared with ITO and aluminum (Al) foil, the surface of Cu plate is rougher. It leads to the adhesive strength of SMSC tape on Cu plate much stronger than those on ITO and Al foil. As shown in the inset of Fig. 4d, SMSC tape (5 × 5 mm^2^) attached on Cu plate can withstand a load of 1.252 kg in the shear direction (corresponding to 0.50 MPa). The site of fracture is located at the interface between SMSC tape and Cu plate, not the SMSC tape. This may be due to the fact that the maximum tensile strength of SMSC tape is much larger than the adhesive strength between Cu plate and SMSC tape [42]. The adhesive strength of SMSC tape exhibits an outstanding fatigue resistance performance. As shown in Fig. S7, there is no obvious degradation in adhesive strength and electric contact between Cu plate and SMSC tape after 500 times of applying-releasing shear load. In addition, this adhesive strength is affected by environmental temperature. As the environmental temperature increases, the adhesive strength of the interface between Cu plate and SMSC tape gradually decreases, along with the decline in its conductivity (Fig. S8). Once the environmental temperature is greater than the melting temperature of SMSC tape, the Cu plate and SMSC tape will be completely separated under a small load. It is worth noting that this thermal fatigue is caused by softening of SMSC tape under high temperature, which is different from the coefficient of thermal expansion mismatch commonly used for interconnecting materials [43, 44].

The content of each component in SMSC tape and annealing temperature can directly affect the intrinsic adhesive strength. Shear adhesive strength of SMSC tape synthesized by different formulations has been compared in Fig. S9. When the mass ratio between SA and UDA changes from 40:60 to 90:10, the adhesive strength increases first and then decreases. Similarly, it also increases first and then decreases with the increase of Ag NPs content in the polymer. On the other hand, with the increase in BPO content and annealing temperature, the shear adhesive strength of SMSC tape decreases. However, both the optimization of component content and the change of annealing temperature essentially change the wetting behavior of SMSC tape in the heating state, thus determining the intrinsic adhesive strength of SMSC tape.

### Potential Applications

**Fig. 5:**
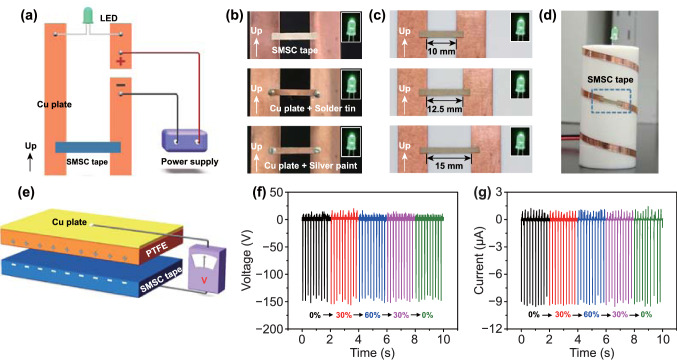
Potential applications of SMSC tape. **a** Schematic illustration of an LED in a simple series circuit powered by a direct-current power supply. **b** Comparison in brightness of the working LEDs, where the circuits are repaired by SMSC tape, Cu plate and solder, and Cu plate and silver paste, respectively. **c** Comparison in brightness of the working LEDs, where the circuits are repaired by SMSC tape with different tensile strain. **d** Optical image of a circuit used to light LED. The circuit is repaired by bending SMSC tape. **e** Schematic illustration of a SMSC tape-based TENG. **f** Output voltage and **g** current of SMSC tape-based TENG when SMSC tape is fixed at different tensile strains in a stretching-releasing cycle

Because of the advantages of stable conductivity, perfect shape memory property and temperature switching reversible adhesion, SMSC tape can independently accomplish the task that requires cooperation of multiple materials or cannot be finished by existing electronic interconnecting materials. In general, a conductor is fixed between two terminals by solder or silver paste to make electric current conduct, while SMSC tape can fix itself on the Cu plate by its own adhesion to conduct the circuit (Fig. 5a). The solution in silver paste and the high temperature required to melt solder present potentially hazards to circuits, especially complex and highly integrated printed circuit board, which challenges the soldering skills of professionals. By contrast, an inexperienced person can easily repair the circuit through SMSC tape. Just a 50–60 °C heating for several seconds (such as offered by a hair dryer) and finger pressing are all that it takes to relight up the light-emitting diode (LED) (Figs. 5b and S10). Meanwhile, SMSC tape is easy to remove from the Cu substrate after reheating and no residue leaves on the substrate (Video S2). Even if the distance between the two Cu plates is larger than the original length of SMSC tape, the circuit still can be easily repaired by stretching SMSC tape (Fig. 5c). SMSC tape is also shaped into a bending pattern on a cylindrical substrate. The bending deformation has no effect on the brightness of the LED (Fig. 5d).

SMSC tape is also able to handle the responsibility of electrode by itself. A SMSC tape-based triboelectric nanogenerator (TENG) working in two-electrode mode is prepared. SMSC tape and polytetrafluoroethylene (PTFE) film are used as negative and positive triboelectric layer, respectively. Moreover, SMSC tape is used as an electrode due to its conductivity. A Cu plate is attached on the back of PTFE film to collect the current from the PTFE film (Fig. 5e). SMSC tape-based TENG driven by manual tapping can light up at least 42 commercial LEDs (Video S3). The triboelectric performance of SMSC tape-based TENG is investigated, when SMSC tape is fixed at different tensile strains in a stretching-releasing cycle. The results show that the output voltage and current (under a stress of ~ 0.2 MPa at a frequency of 5 Hz) are almost unchanged (Fig. 5f, g). These experimental results indicate that SMSC tape is a promising candidate as a novel conductive connecting material for applications in the next generation of electronic devices in the future.

## Conclusion

In summary, we designed a highly stretchable shape memory self-soldering conductive tape with reversible adhesion switched by temperature. The tape is prepared by anaerobic curing of well blended precursors, including SA, UDA, BPO and Ag NPs. A series of experimental results show that SMSC tape exhibits an initial conductivity of ~ 2772 S cm^−1^ and a maximum strain of ~ 100%. With the increase in Ag NPs content, the initial conductivity could be increased to 5446 S cm^−1^ by decreasing the strain to 17%. Meanwhile, SMSC tape has the perfect shape memory performance and fatigue resistance ability. The perfect shape memory effect gives SMSC tape a temperature-determined reversible strong–weak adhesion transition. The adhesive strength of SMSC tape can be compared with the commercial conductive tape, where the adhesive mechanism is from the embedded structure rather than the molecular interaction. Thanks to the stable conductivity, perfect shape memory performance and reversible self-adhesion, SMSC tape can serve two functions of electrode and solder simultaneously. As a new type of conductive interconnecting material, SMSC tape possesses the characteristics of no adhesive residue and low processing temperature, and has promising compatibility with modern electronic devices. It also provides a unique idea for the development of electronic devices in the future.

## Supplementary Information

Below is the link to the electronic supplementary material.

Supplementary file1 (PDF 514 kb)

Supplementary file2 (MP4 13955 kb)

Supplementary file3 (MP4 11015 kb)

Supplementary file4 (MP4 4612 kb)
