# Supplementary material for: Highly Stretchable Shape Memory Self-Soldering Conductive Tape with Reversible Adhesion Switched by Temperature
Source: Nanomicro Lett. 2021 May 11;13:124. doi: 10.1007/s40820-021-00652-0 (PMC8113436; doi:10.1007/s40820-021-00652-0)
Supplement: Supplementary file 1 — Supplementary file1 (PDF 514 kb) [file 40820_2021_652_MOESM1_ESM.pdf]

Supporting Information for

Highly Stretchable Shape Memory Self-Soldering Conductive Tape with Reversible Adhesion Switched by Temperature

Mengyan Wang[1], Quan Zhang[1], Yiwen Bo[1], Chunyang Zhang[1], Yiwen Lv[1], Xiang Fu[1], Wen He[1], Xiangqian Fan[1], Jiajie Liang[1], Yi Huang[1], Rujun Ma[1,]*, Yongsheng Chen[2]

[1]School of Materials Science and Engineering, National Institute for Advanced Materials, Tianjin Key Lab for Rare Earth Materials and Applications, Nankai University, Tongyan Road 38, Tianjin, 300350 P. R. China

[2]State Key Laboratory and Institute of Elemento-Organic Chemistry, Centre of Nanoscale Science and Technology and Key Laboratory of Functional Polymer Materials, College of Chemistry, Nankai University, Weijin Road 94, Tianjin, 300071 P. R. China

Mengyan Wang and Quan Zhang contributed equally to this work.

*Corresponding author. E-mail: malab@nankai.edu.cn (Rujun Ma)

Supplementary Tables and Figures

Table S1 Comparison of melting temperature and mechanical properties between SA-UDA copolymer and SMSC tape. The mass ratio between SA and UDA is fixed at 70:30

Materials	T_m (°C)	Elastic modulus (at 60 °C) (MPa)	Maximum stress (at 60 °C) (MPa)	Maximum tensile strain (at 60 °C) (%)	Elastic modulus (at room temperature) (MPa)	Maximum stress (at room temperature) (MPa)	Maximum tensile strain (at room temperature) (%)
SA-UDA copolymer	41.0	0.025	0.6	395	35.7	4.0	41
SMSC tape	42.4	0.36	0.2	93	52.2	3.7	10

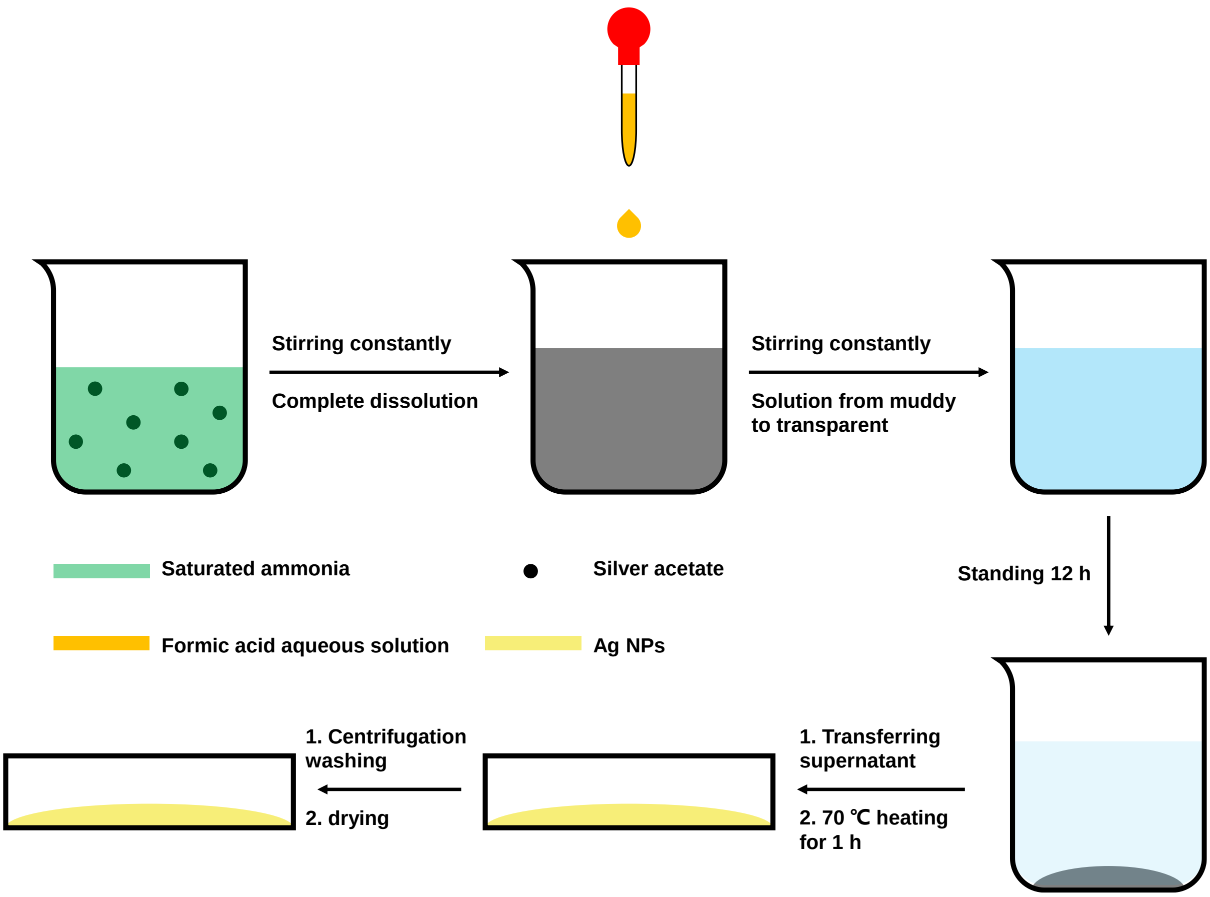

Fig. S1 Synthesis of thermal reduced Ag NPs

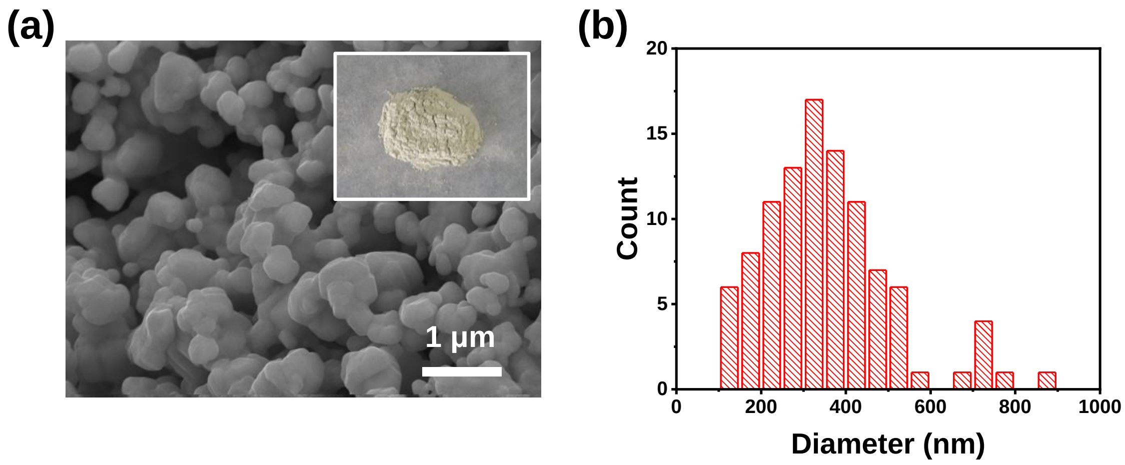

Fig. S2 Characterization of thermal reduced Ag NPs. **a** Morphology SEM image of thermal reduced Ag NPs. The scale bar is 1 μm. The inset is the optical image of Ag NPs. **b** Size distribution of thermal reduced Ag NPs. Particle-size measurement indicates that the diameter of NPs is bimodal distribution, with the broad distribution centered at ~300 nm and ~700 nm, respectively.

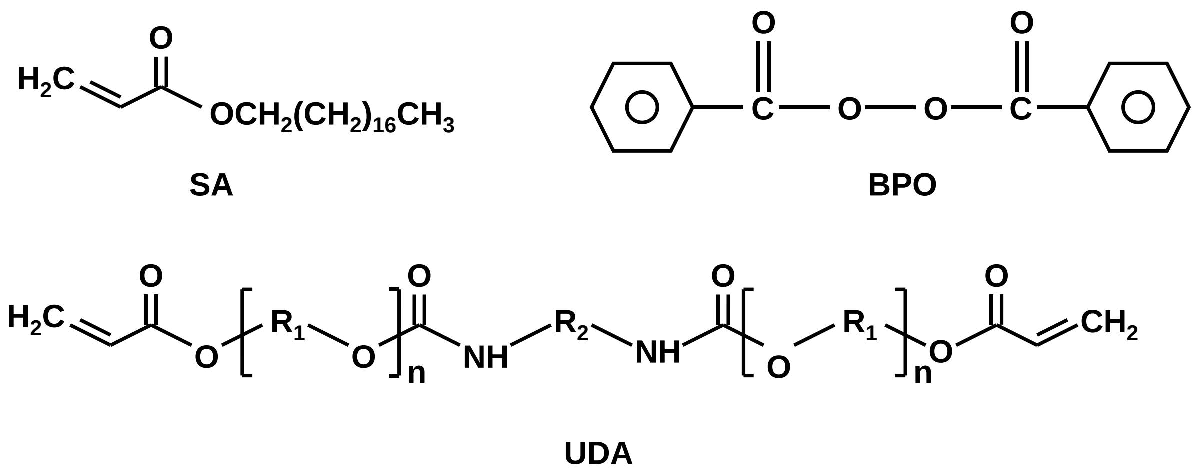

Fig. S3 Chemical structures of monomers (SA and UDA) and radical initiator (BPO)

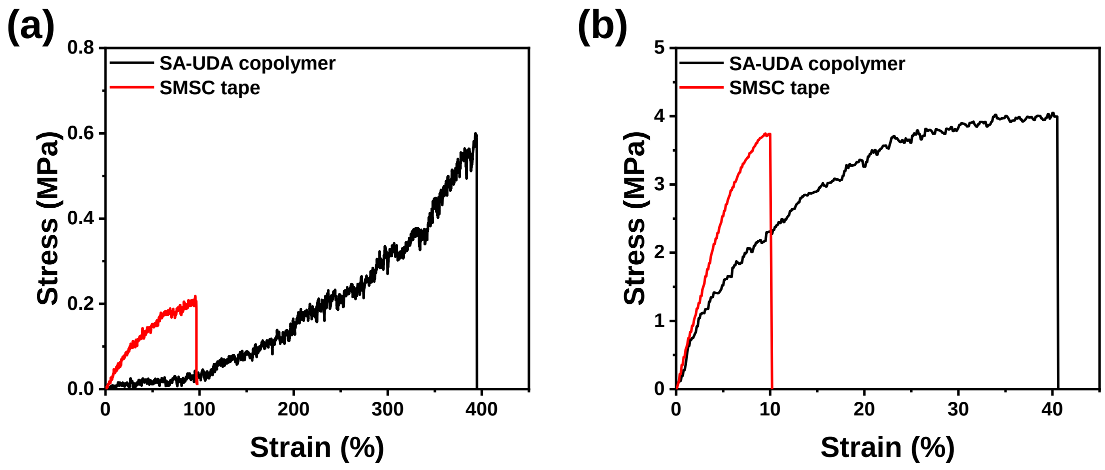

Fig. S4 Stress of SA-UDA copolymer and SMSC tape as a function of strain at **a** 60 °C and **b** room temperature. The calculated elastic modulus and maximum stress are listed in Table S1.

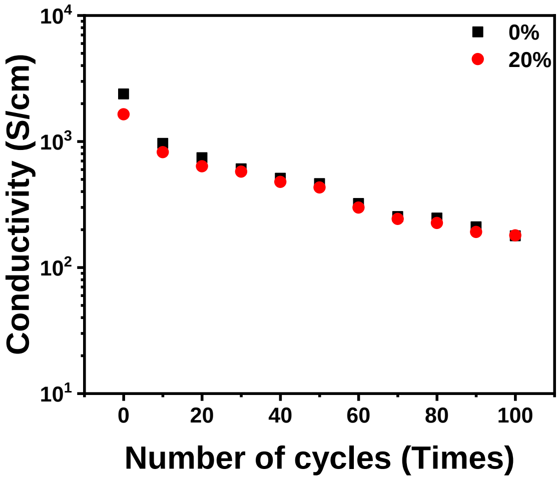

Fig. S5 Conductivity of SMSC tape as a function of the shape memory stretching cycles. The maximum tensile strain was 20%

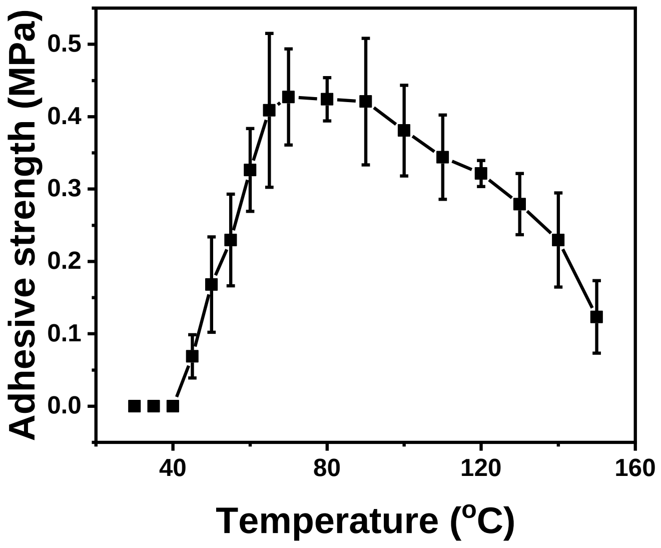

Fig. S6 Shear adhesive strength between SMSC tape and Cu plate at room temperature as a function of heating temperature. When the temperature is lower than the melting temperature of SMSC tape, the shear adhesive strength is almost zero. As the temperature gradually increases, the shear adhesive strength significantly increases and gradually becomes stable. With further increase of heating temperature, the shear adhesive strength decreases because of the over-softening of SMSC tape.

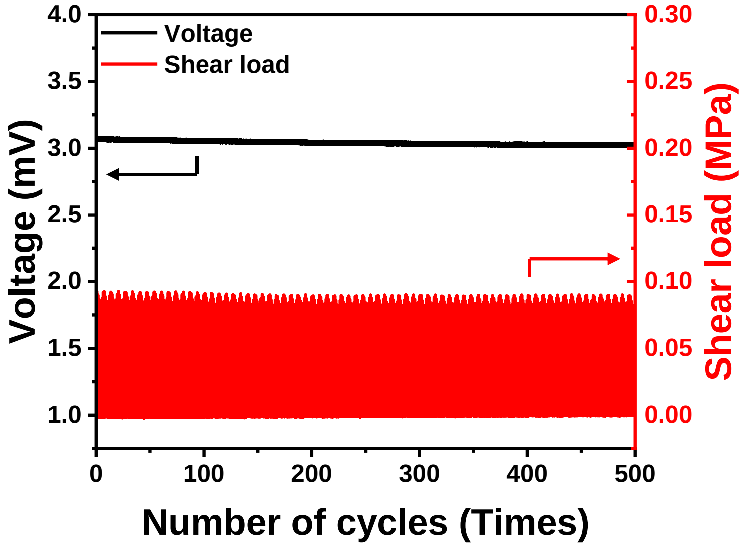

Fig. S7 Voltage applied on Cu plate-SMSC tape-Cu plate sandwich structure under repeated applying-releasing shear load. The current is always 1 mA and the tensile distance is constant.

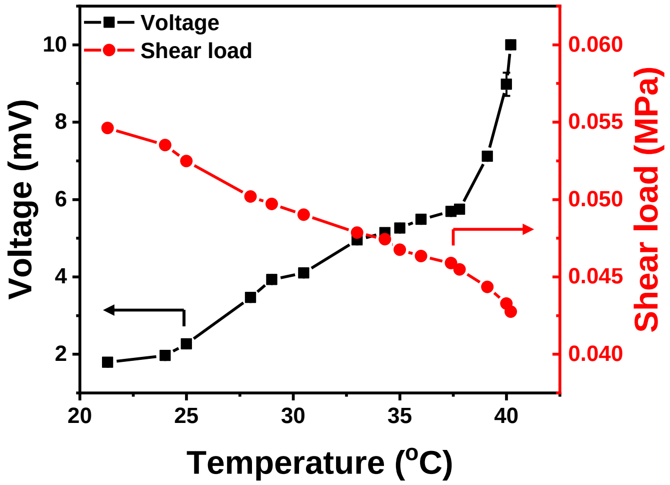

Fig. S8 Effect of environmental temperature on adhesive strength and voltage between Cu plate and SMSC tape. The current is always 1 mA and the tensile distance is constant.

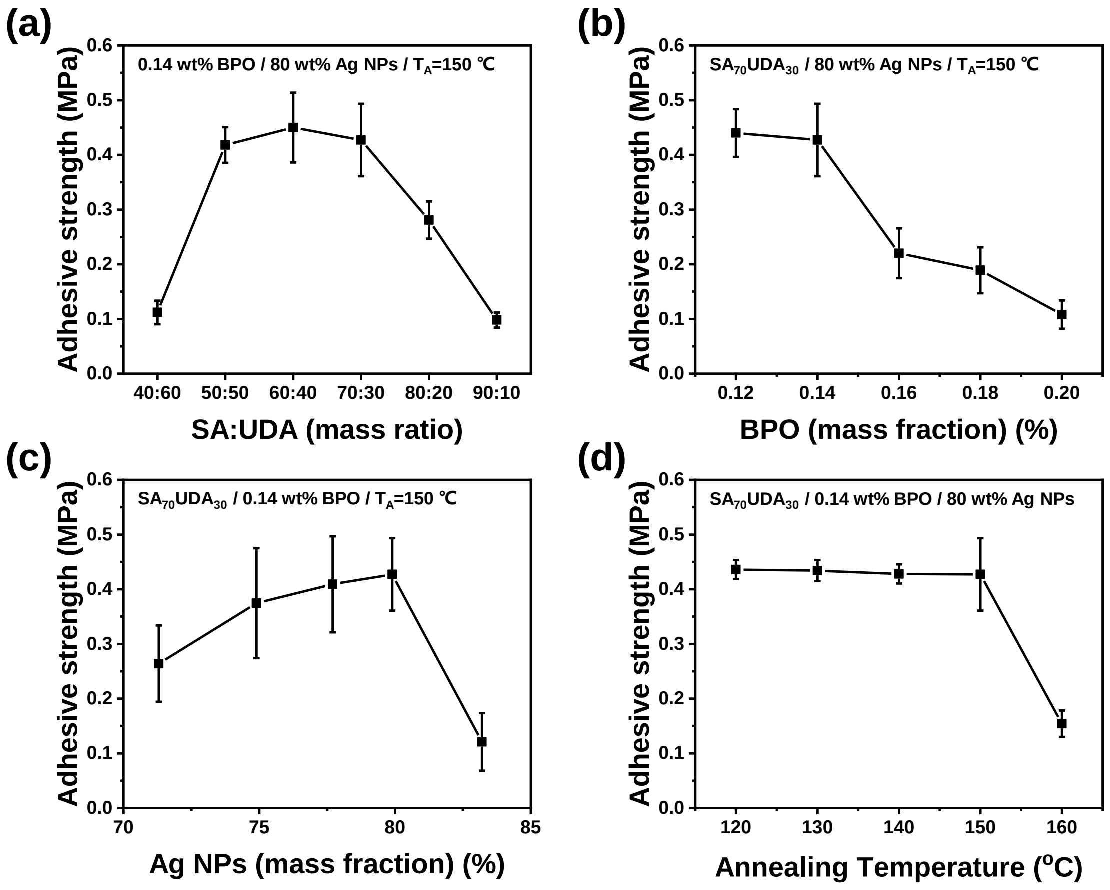

Fig. S9 Adhesive strength of SMSC tape under different four parameters, including **a** weight ratio between SA and UDA, **b** content of BPO, **c** content of Ag NPs and **d** annealing temperature, on shear adhesive strength at cooling temperature. The heating temperature is 60 °C and the pressing stress is ~0.36 MPa. In each contrast experiment, the rest three parameters are fixed.

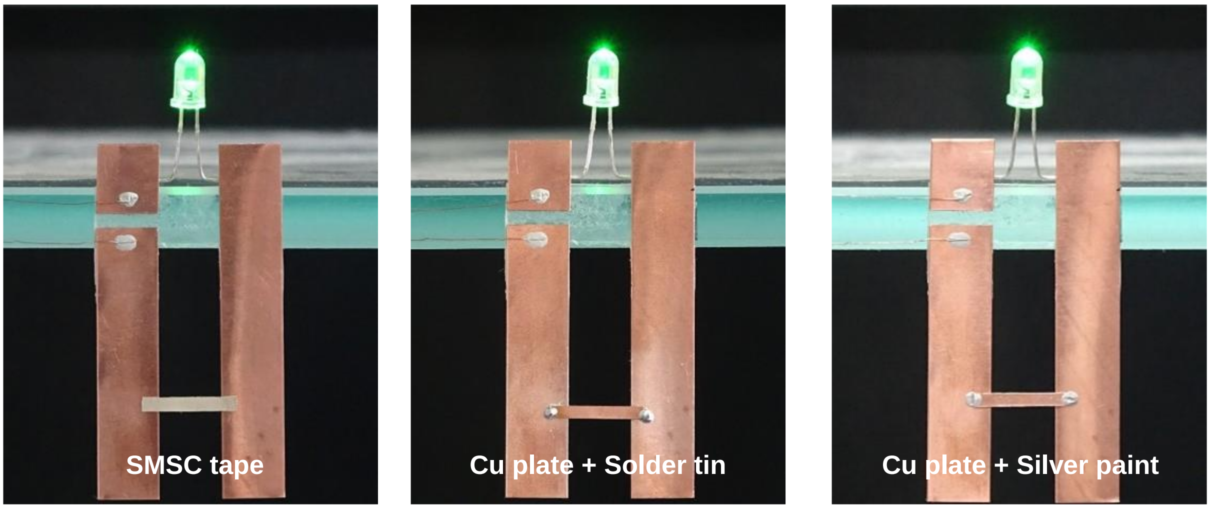

Fig. S10 Optical Images of the circuits repaired by SMSC tape, Cu plate and solder, and Cu plate and Ag adhesive
